# Research on design and control method of active vibration isolation system based on piezoelectric Stewart platform

**DOI:** 10.1038/s41598-024-84980-2

**Published:** 2025-01-06

**Authors:** Zhiyi Fang, Zhiliang Yu, Qingping Huang, Yanfen Wang, Xingsheng Gu

**Affiliations:** 1 East China University of Science and Technology, Shanghai, China; 2 Aerospace System Engineering Shanghai, Shanghai, 201100 China; 3 National Key Laboratory of Aerospace Mechanism, Shanghai, China

**Keywords:** Active vibration isolation, Piezoelectric actuator, Hysteresis, Stewart platform, Linearization, Engineering, Materials science

## Abstract

Space payloads in orbit are vulnerable to small vibrations from satellite platforms, which can degrade their performance. Traditional methods typically involve installing a passive vibration isolation system between the platform and the payload. However, such systems are usually effective only for high-frequency, large-amplitude vibrations and perform poorly in isolating low-frequency vibrations and resonances below 10 Hz. To address this limitation, this paper proposes an active vibration isolation system using a 6-degrees-of-freedom Stewart platform driven by piezoelectric actuators. First, the characteristics of the Stewart platform are analyzed and modeled, with the deformation displacement of each leg calculated through decoupling, allowing for high-precision servo control. Next, given the inherent hysteretic nonlinearity of piezoelectric ceramics, which significantly affects positioning accuracy, the hysteresis mechanism of the actuators is analyzed, and a phenomenological mathematical model based on Bouc–Wen operators is established. A Modified particle swarm optimization (MPSO) method is proposed for identifying the model’s nonlinear parameters, significantly enhancing the optimization efficiency. Finally, feedforward inverse compensation and feedback linearization methods are introduced. Experimental results verify that the designed active–passive vibration isolation system greatly improves both the positioning accuracy of the piezoelectric actuators and the active vibration isolation performance of the platform.

## Introduction

With the advancement of aerospace technology, the political and military sectors of various countries have placed higher demands on satellite communications^1–3^, Earth observation, and antenna adjustment technologies. Consequently, the performance requirements for space payloads have also increased^4–7^. Spacecraft in orbit frequently encounter various disturbances, such as solar panel vibrations, high-speed rotational forces from control torque gyroscopes, and instrument operations, collectively known as spacecraft micro-vibrations^8–11^. These micro-vibrations are typically characterized by low frequencies, generally below 10 Hz, and small amplitudes in the micro-arc range. While these disturbances minimally affect most spacecraft payloads, they significantly impact certain sensitive instruments, such as laser communication terminals high-precision remote sensing payloads, and space cameras, potentially degrading resolution and stability^12–14^. In severe cases, system malfunctions may occur^15–17^.

Currently, the primary method for suppressing micro-vibrations is passive vibration isolation, which employs a spring-damping system between the platform and payload. This system absorbs and dissipates vibration energy through a combination of elasticity and damping^18,19^. Passive systems are simple, energy-independent, autonomous, and highly reliable, making them suitable for large-scale deployment. However, passive isolation primarily focuses on stiffness and damping. The inclusion of negative stiffness technology can complicate the system structure, and these systems are more effective at suppressing high-frequency, large-amplitude vibrations but perform poorly for low-frequency micro-vibrations.

In contrast, active vibration isolation systems use external actuators and controllers^20–22^. These systems overcome the limitations of passive isolation by incorporating external energy and a complete feedback loop, enabling high-precision vibration isolation. Active systems typically include actuator units, sensor units, data processing systems, control systems, and power supplies.

The Stewart platform, with its high stiffness-to-mass ratio, precision, and multi-degree-of-freedom capabilities, is well-suited for applications requiring precise motion control, particularly for sensitive space payloads. Its six degrees of freedom make it an ideal candidate for active vibration isolation systems. However, the performance of the Stewart platform heavily depends on its actuators. Current actuators include piezoelectric actuators, voice coil motors, magneto strictive actuators, and shape memory alloys. Among these, piezoelectric actuators stand out for their compact size, high output force, precision, wide bandwidth, and fast response, making them the optimal choice. However, the nonlinear hysteresis between the input and output of piezoelectric ceramics can affect positioning accuracy and, in severe cases, lead to system instability. Therefore, the study and analysis of piezoelectric actuators are essential.

Based on the above analysis, this paper proposes an active vibration isolation system using a Stewart platform. By analyzing the platform’s characteristics, a dynamic model is established, and the displacement and deformation of each leg are decoupled. A Bouc–Wen-based mathematical model is developed to represent the hysteresis of the piezoelectric actuators. To address the nonlinear parameter identification issue, an improved Modified Particle Swarm Optimization algorithm is introduced^23–26^, which enhances identification efficiency and mitigates the problem of falling into local minima during optimization. Building on this, a control method combining feedforward inverse compensation linearization and feedback is proposed to improve control accuracy in the active vibration isolation system. Finally, an experimental setup is developed to validate the proposed system. The results demonstrate the system’s effectiveness in achieving high-precision positioning and offer valuable insights for future applications in fields requiring precise control, such as large controllable antennas, active vibration isolation for satellite platforms, and sensitive laser communication payloads.

In this paper, the method of the control outputs of the Stewart six-degree-of-freedom platform are first decoupled into six piezoelectric actuators is proposed. The methods of the hysteresis of each actuator is accurately modeled and controlled via feedback, with sky-hook control ultimately applied to the entire Stewart active vibration isolation platform are proposed.

## Piezoelectric actuator hysteresis modeling and parameter identification

Currently, the mathematical models used to describe hysteresis mainly rely on phenomenological models, which are based on the relationship between the input and output of actuators. Examples of such models include the Preisach model, the Prandtl–Ishlinskii model, the Bouc–Wen model, and the Maxwell model, among others. The Bouc model of hysteresis, later modified by Wen, was initially developed for nonlinear vibrational mechanics. It has since been widely applied in structural and mechanical engineering. This model is characterized by a set of two nonlinear differential equations that relate the applied mechanical force *f* to the deformation *x* of a structure.

### Hysteresis modeling

The displacement control of the Stewart platform is achieved using piezoelectric actuators, and the method for controlling hysteresis mainly involves two key aspects. The first aspect is the development of an accurate mathematical model of hysteresis, as the model’s precision directly affects the system’s ability to describe hysteresis behavior. The second aspect is the accuracy of parameter identification within the model. Since the parameters have a critical influence on the model, large parameter deviations can result in the hysteresis model failing to accurately describe the behavior. This also impacts inverse compensation, leading to significant deviations in the predicted compensation voltage. Therefore, both modeling and parameter identification are essential. The following section will analyze hysteresis modeling. The output of a piezoelectric actuator can be expressed using the following formula:

1 $$Hr(t) = \left\{ \begin{gathered} x(t) = \alpha ku(t) + (1 - \alpha )kz(t) \hfill \\ \dot{z} = A\dot{x} - \beta \left| {\dot{x}} \right|\left| z \right|^{n - 1} z - \lambda \dot{x}\left| z \right|^{n} \hfill \\ \end{gathered} \right.$$

where $$\alpha$$ controls the restoring force amplitude, $$\alpha = rand()$$$$\alpha \in (0,1)$$, $$A$$, $$\beta$$, $$\lambda$$ are the dimensionless parameters; *n* controls the smoothness of the transition from elastic to plastic response; $$x(t)$$ is the displacement output, which $$z(t)$$ is the intermediate quantity. The Bouc–Wen model has the characteristics of simple form and few identification parameters, which has strong applicability for the design of the controller in practical applications, and its code is easy to implement in embedded systems.

**Fig. 1:**
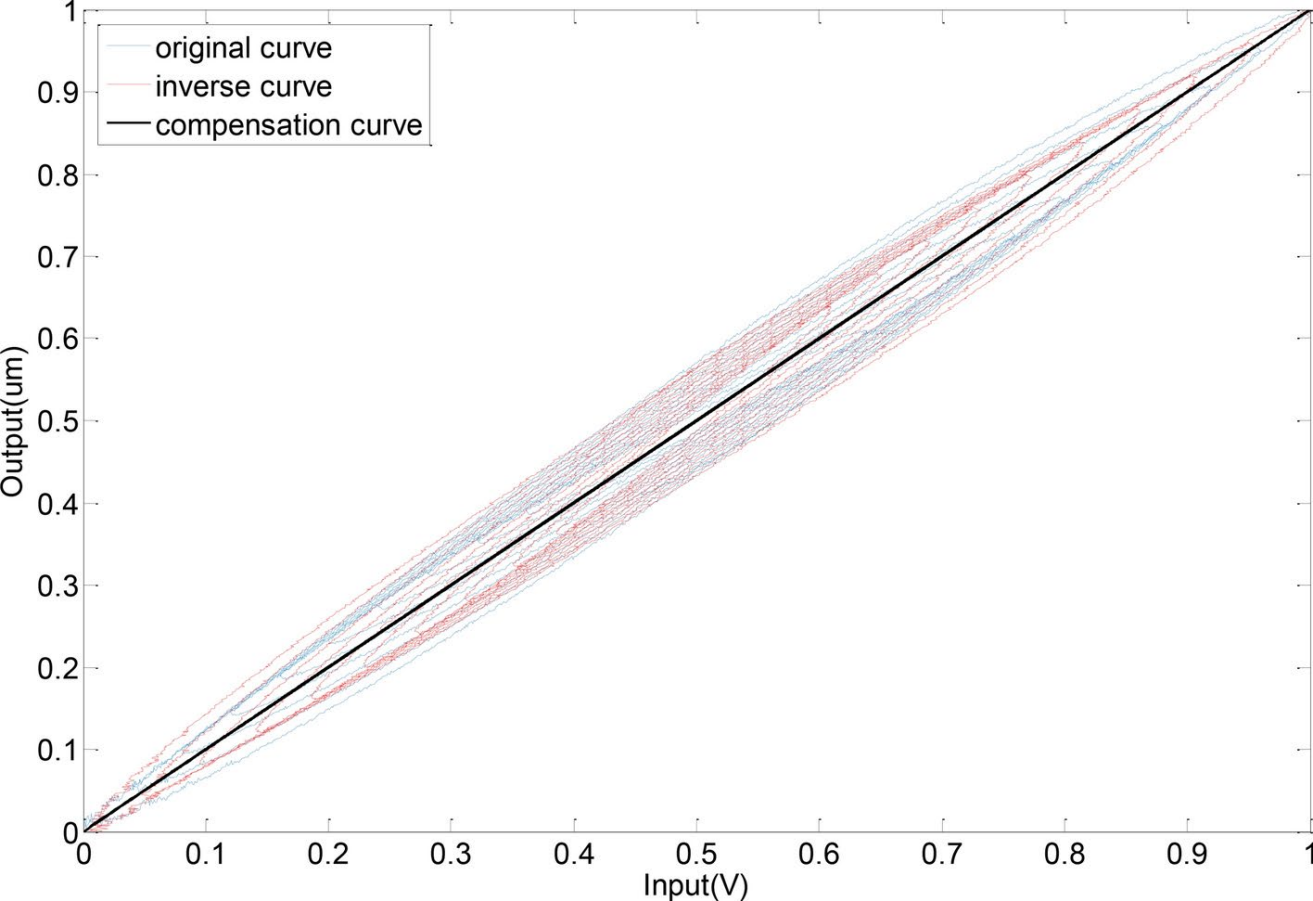
Original hysteresis curve.

**Fig. 2:**
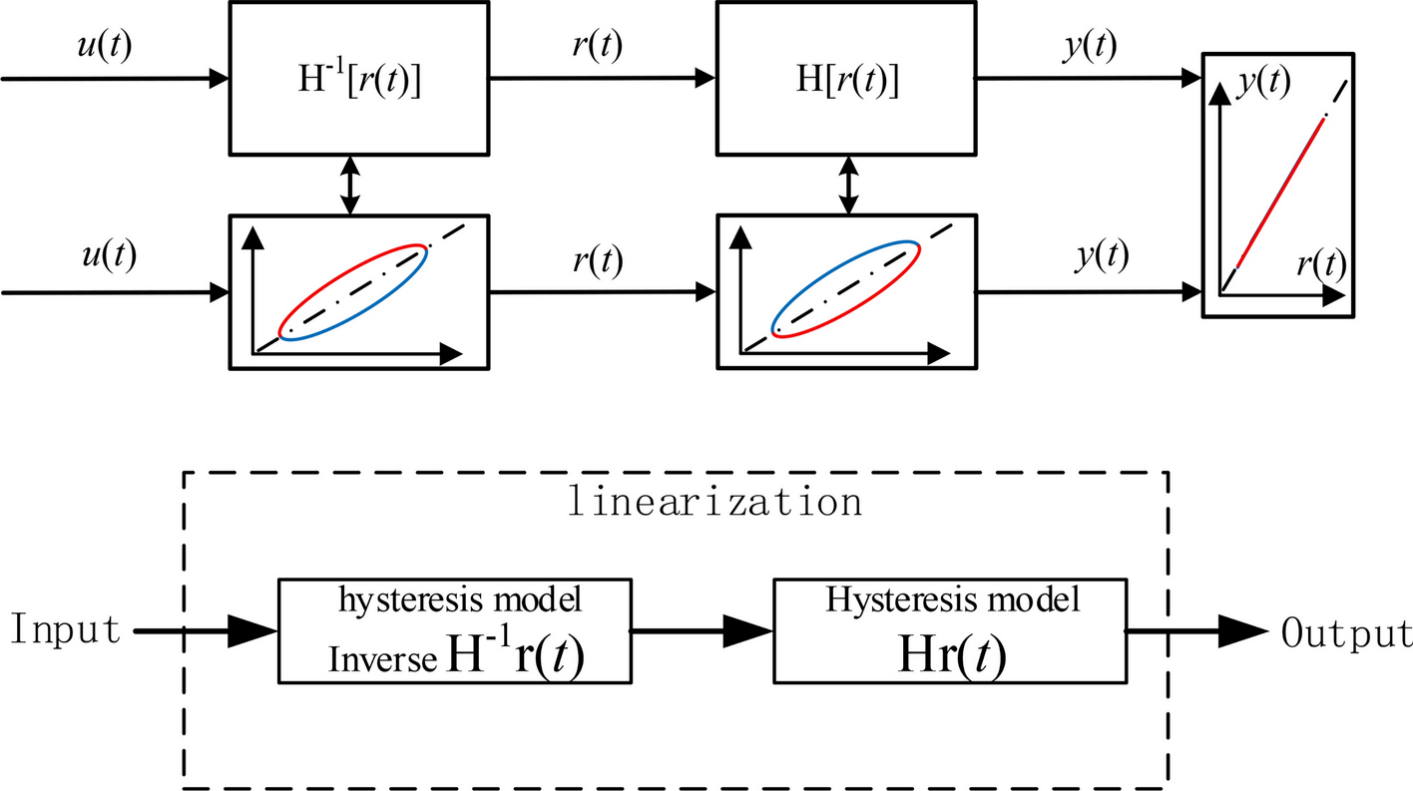
Inverse hysteresis compensation.

Feed-forward linearization process as shown in the Figs. 1 and 2, in the Fig. 1 the original hysteresis curve (blue original curve) and the hysteresis curve of the inverse compensation (red inverse curve) have a symmetric relationship of y = x (black curve), and the hysteresis inverse compensation method is established according to this relationship. The expected input signal *u*(*t*) is converted into an intermediate control input signal *r*(*t*) by the inverse hysteresis model, where the anti-hysteresis relationship between the expected input and the control signal is presented, and the intermediate control input signal acts on the piezoelectric ceramic actuator to produce displacement, due to the anti-hysteresis effect, cancels the positive hysteresis effect of the piezoelectric ceramic actuator, and finally the actual output *y*(*t*) and the expected input *u*(*t*) present a linear relationship, thereby reducing or eliminating the hysteresis.

2 $$H^{ - 1} r(t) = \left\{ \begin{gathered} r(t) = \alpha ku(t) + (1 - \alpha )kz(t) \hfill \\ \dot{z} = A\dot{r} - \beta \left| {\dot{r}} \right|\left| z \right|^{n - 1} z - \lambda \dot{r}\left| z \right|^{n} \hfill \\ \end{gathered} \right.$$

### Improved MPSO parameter identification

**Fig. 3:**
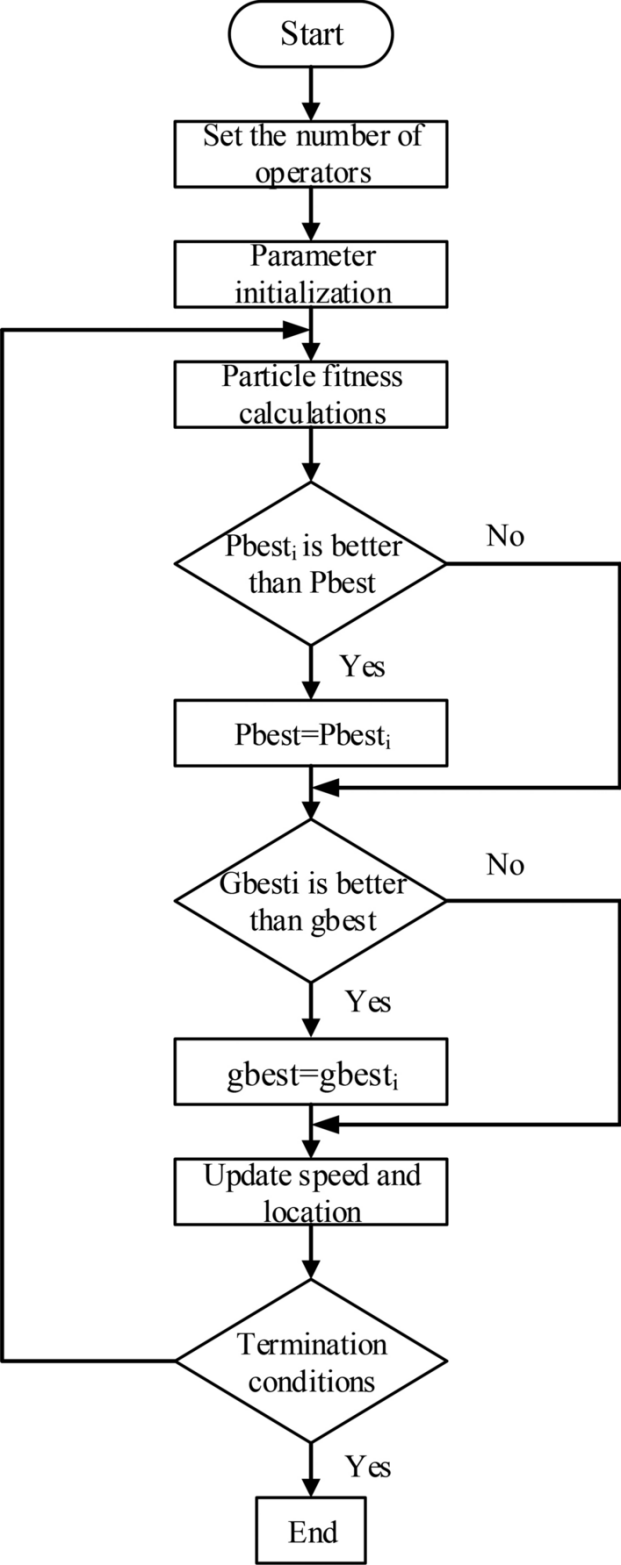
The algorithm flow of MPSO.

Since the parameters in the Bouc–Wen model are nonlinear, it is challenging to identify them using conventional linear methods. Therefore, this paper employs an improved Modified Particle Swarm Optimization (MPSO) method to identify the parameters in the model as shown in the Fig. 3. The target fitness function is chosen as follows:

3 $$f(X) = \frac{1}{N}\sum\limits_{i = 1}^{N} {(y_{i} - \tilde{y}_{i} )}$$

There $N$ is the number of data points, $y_{i}$ is the output values for the model, $\tilde{y}_{i}$ is the actual values.

The traditional Particle Swarm Optimization (PSO) method faces the issue of particles becoming trapped in local optima, preventing the algorithm from finding the global optimal solution. To address this, an improved velocity update method is proposed in this paper. Based on the traditional PSO algorithm, the velocity and position update equations are as follows:

4 $$\left\{ \begin{gathered} V_{i}^{t + 1} = \omega V_{i}^{t} + c_{1} r_{1}^{t} (P_{besti}^{t} - X_{i}^{t} ) + \cdots \hfill \\ c_{2} r_{2}^{t} (G_{best}^{t} - X_{i}^{t} ) \hfill \\ X_{i}^{t + 1} = X_{i}^{t} + V_{i}^{t + 1} \hfill \\ \end{gathered} \right.$$

where $r_{i}^{t} = rand_{i} (),i = 1,2, \ldots$ is a random number, $\omega$ is a particle swarm inertia factor, $c_{1}$ isa local acceleration coefficient and $c_{2}$ is a global acceleration coefficient, respectively. The inertia weight factor influences the overall particle search behavior: a large inertia factor facilitates a broad global search, while a small inertia factor is more conducive to a fine-tuned local search. In traditional MPSO, a constant inertia factor is typically used, which imposes limitations on the search process. To overcome this, this paper replaces the constant inertia weight with a dynamic inertia weight.

5 $$\omega_{i} = \omega_{\min } \exp (\frac{{\omega_{\max } - \omega_{\min } }}{T}(T - t_{i} ))$$

where $i = 1,2, \cdots ,T$, $\omega_{\max }$ is the maximum weight factor, $\omega_{\min }$ is the minimum value of the weight factor, $t_{i}$ represents the *ith* particle, and $T$ is the number of iterations. Improved dynamic weight factor formula can be expressed as

6 $$\begin{gathered} V_{i}^{t + 1} = \omega_{\min } \exp (\frac{{\omega_{\max } - \omega_{\min } }}{T}(T - t_{i} ))V_{i}^{t} + c_{1} r_{1}^{t} (P_{besti}^{t} - X_{i}^{t} ) + \cdots \hfill \\ c_{2} r_{2}^{t} (G_{best}^{t} - X_{i}^{t} ) + c_{3} r_{3}^{t} (Q_{besti}^{t} - X_{i}^{t} ) \hfill \\ \end{gathered}$$

$c_{3}$ is a correlation acceleration coefficient, $r_{3}^{t}$ is a random number between 0 and 1, $Q_{besti}^{t}$ is a randomly selected historical position information. The parameter identification process is as follows.

*Step 1*: Set the operator population;

*Step 2*: Initialize the particle parameters, set the population size, number of iterations, acceleration coefficient, and other initialization values;

*Step 3*: Initialize the velocity and position of each particle in the population;

7 $$\left\{ \begin{gathered} X_{i} = X_{\min } + {\text{rand}}(x)(X_{\max } - X_{\min } ) \\ V_{i} = V_{\min } + {\text{rand}}(x)(V_{\max } - V_{\min } ) \\ \end{gathered} \right.$$

*Step 4*: Calculate particle fitness according to step 3;

*Step 5*: Look for individual optimal and global optimal positions;

8 $$\begin{gathered} pbest_{i} (t + 1) = \hfill \\ \left\{ \begin{gathered} pbest_{i} (t),f(X_{i} (t + 1)) \ge f(pbest_{i} ) \hfill \\ X_{i} (t + 1)),f(X_{i} (t + 1)) < f(pbest_{i} ) \hfill \\ \end{gathered} \right. \hfill \\ \end{gathered}$$

9 $$gbest(k + 1) = \min (pbest_{i} (k + 1))$$

*Step 6*: Update the speed and position according to Eqs. (8) and (9);

*Step 7*: Determine the termination conditions, if the termination conditions are met, the optimization process will be stopped, and if it is not met, return to step 4 to continue the optimization.

## Design and control method of active vibration isolation system

### Platform kinematics inverse solution

The active vibration isolation system is based on a six-degrees-of-freedom Stewart platform, using piezoelectric ceramic actuators as the platform’s outriggers. The system employs a controller to drive each actuator, achieving high-precision vibration isolation for both the upper and lower platforms^27–30^.

The parameter design of the six-degrees-of-freedom platform and the development of the motion control system necessitate kinematic analysis of the mechanism. This involves establishing a mathematical model for input–output position analysis, which reveals the relationship between the telescopic displacement of the piezoelectric actuators and the position and orientation of the motion platform. The kinematic inverse solution is used to determine the joint coordinates from the working coordinates, thereby deriving the telescopic displacement (unique solution) that each actuator should output for a given platform position. Consequently, a kinematic simplified model of the six-degrees-of-freedom pointing mechanism is developed.

**Fig. 4:**
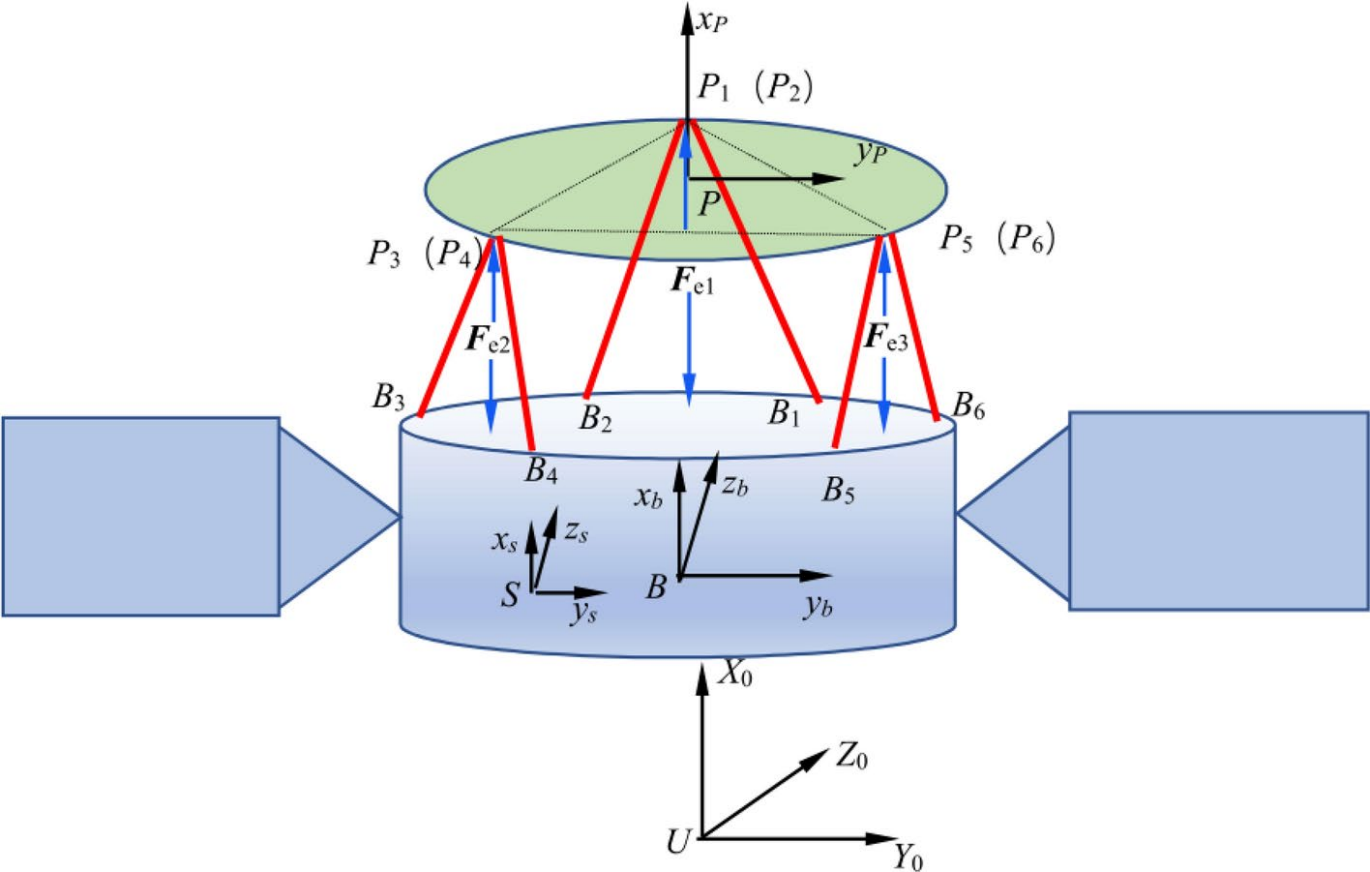
The 6 degrees of freedom platform coordinate system.

Based on the geometric vector relationship between the fixed coordinate system B-XYZ and the moving coordinate system P-xyz, the vector expression of the outriggers is obtained as shown in the Fig. 4.

10 $$\overrightarrow {{B_{i} P_{i} }} = \overrightarrow {BP} + \overrightarrow {{PP_{i} }} - \overrightarrow {{BB_{i} }}$$

The displacement output platform (moving platform) has two key parameters in the fixed coordinate system: position and attitude. The position is represented by the coordinates (x, y, z) in the fixed coordinate system B-XYZ, which corresponds to the center of the moving platform. The attitude of the moving platform is described by the rotation angles around the coordinate axes: α\alphaα for rotation around the x-axis, β for rotation around the y-axis, and γ for rotation around the z-axis. The attitude is expressed as the Euler angles (α, β, γ). The rotation transformation matrix of the moving coordinate system P-xyz relative to the fixed coordinate system B-XYZ is used to represent this attitude.

11 $$\begin{aligned} R & = R_{x} (\alpha )R_{y} (\beta )R_{z} (\gamma ) \\ & = \left[ {\begin{array}{*{20}l} {\cos (\beta )\cos (\gamma )} \hfill & { - \cos (\beta )\sin (\gamma )} \hfill & {sin(\beta )} \hfill \\ {\cos (\gamma )\sin (\alpha )sin(\beta ) + \cos (\alpha )sin(\gamma )} \hfill & {\cos (\alpha )\cos (\gamma ) - \sin (\alpha )\sin (\beta )\sin (\gamma )} \hfill & { - \cos (\beta )\sin (\alpha )} \hfill \\ { - \cos (\alpha )\cos (\gamma )\sin (\beta ) + \sin (\alpha )\sin (\gamma )} \hfill & {\cos (\gamma )\sin (\alpha ) + \cos (\alpha )\sin (\beta )\sin (\gamma )} \hfill & {\cos (\alpha )\cos (\beta )} \hfill \\ \end{array} } \right] \\ \end{aligned}$$

According to the geometric relationship, if the distance between the upper hinge circle radius R_b_, the near hinge center angle *θ*_b_ and the upper hinge point to the moving platform coordinate plane in the moving platform is known, the coordinate vector matrix B of the hinge pivot *P*_i_ (i = 1, 2,…,6) on the platform in the body coordinate system can be determined as

12 $$\begin{aligned} P & = \left[ {\begin{array}{*{20}c} { - R_{b} \sin (\frac{{\theta_{b} }}{2})} & {R_{b} \sin (\frac{{\theta_{b} }}{2})} & {R_{b} \cos (30^{^\circ } - \frac{{\theta_{b} }}{2})} & \cdots \\ { - R_{b} \cos (\frac{{\theta_{b} }}{2})} & { - R_{b} \cos (\frac{{\theta_{b} }}{2})} & {R_{b} \sin (30^{^\circ } - \frac{{\theta_{b} }}{2})} & \cdots \\ { - h_{1} } & { - h_{1} } & { - h_{1} } & \cdots \\ \end{array} } \right. \\ & \quad \left. {\begin{array}{*{20}c} {R_{b} \cos (30^{^\circ } + \frac{{\theta_{b} }}{2})} & { - R_{b} \cos (30^{^\circ } + \frac{{\theta_{b} }}{2})} & { - R_{b} \cos (30^{^\circ } - \frac{{\theta_{b} }}{2})} \\ {R_{b} \sin (30^{^\circ } + \frac{{\theta_{b} }}{2})} & {R_{b} \sin (30^{^\circ } + \frac{{\theta_{b} }}{2})} & {R_{b} \sin (30^{^\circ } - \frac{{\theta_{b} }}{2})} \\ { - h_{1} } & { - h_{1} } & { - h_{1} } \\ \end{array} } \right] \\ \end{aligned}$$

Then, based on the coordinate transformation relationship, the coordinate vector matrix of the hinge pivot points Pi (where i = 1, 2…,6) on the dynamic platform in the fixed coordinate system can be determined as follows:

13 $$\left[ {PP_{i} } \right] = RP$$

The expression for the length vector between two hinge fulcrums is that

14 $$\left[ {l_{i} } \right] = [\begin{array}{*{20}c} y & z & {(x + h_{0} )]^{T} } \\ \end{array} + RP_{i}$$

Therefore, the amount of displacement that the piezoelectric stepper actuator should be stretched and contracted

15 $$\Delta l = \sqrt {(x^{\prime}_{i} - x{\kern 1pt}_{i} )^{2} + (y^{\prime}_{i} - y{\kern 1pt}_{i} )^{2} + (z^{\prime}_{i} - z{\kern 1pt}_{i} )^{2} } - l_{0}$$

### Equivalent model of each unit of the platform

#### One-lever dynamic equivalent modeling

**Fig. 5:**
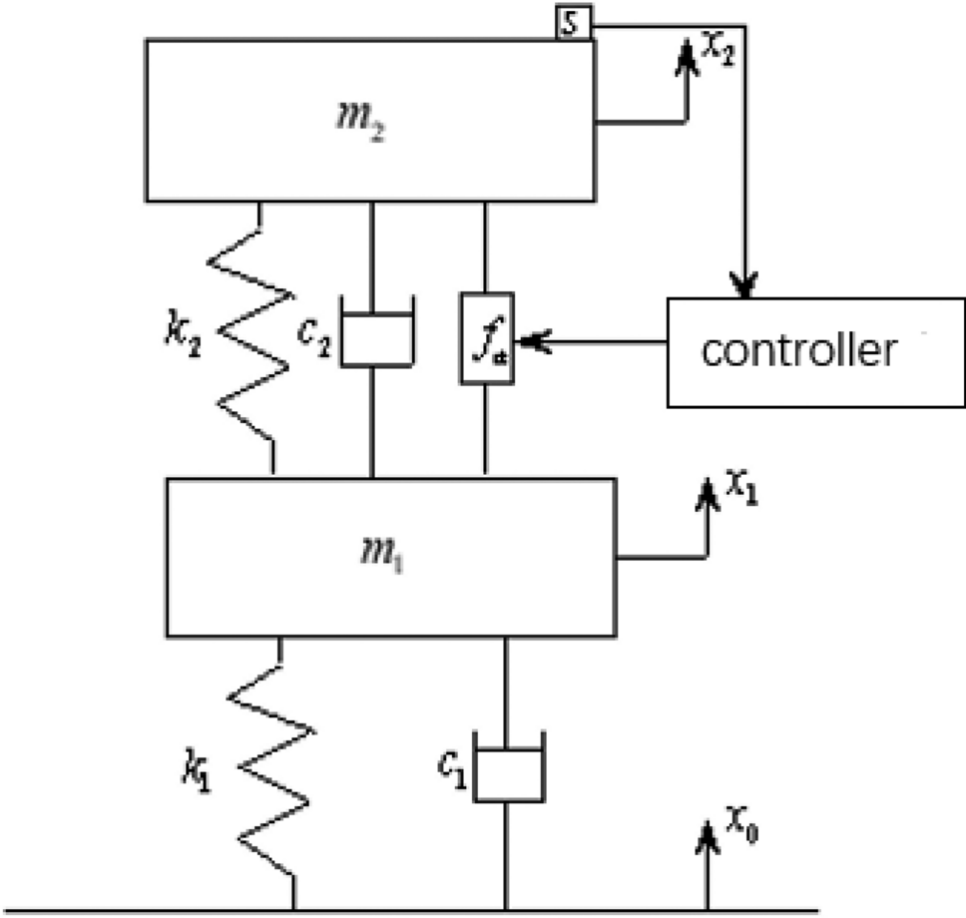
System mechanics diagram.

The single-lever dynamic model of the active–passive integrated actuator, based on piezoelectric ceramics, can be simplified into a hybrid vibration isolation system with two degrees of freedom as shown in the Fig. 5.

16 $$\begin{gathered} m_{1} \ddot{x}_{2} + c_{2} (\dot{x}_{2} - \dot{x}_{1} ) + k_{2} (x_{2} - x_{1} ) = f_{a} \hfill \\ m_{1} \ddot{x}_{1} + c_{1} (\dot{x}_{1} - \dot{x}_{0} ) + c_{2} (\dot{x}_{1} - \dot{x}_{2} ) + k_{1} (x_{1} - x_{0} ) + k_{2} (x_{1} - x_{2} ) = - f_{a} \hfill \\ \end{gathered}$$

$m_{1}$ is the mass of the piezoelectric actuator mechanism; $m_{2}$ is the mass of the shock-isolated load; $c_{1}$ is the damping for eddy current dampers; $c_{2}$ is the damping of piezoelectric actuators; $k_{1}$ is the shrapnel stiffness for eddy current dampers; $k_{2}$ is the equivalent dynamic stiffness for piezo actuators; $x_{0}$ is excitation displacement based on the underlying disturbance; $x_{1}$ is displacement for piezo actuators; $x_{2}$ is the payload displacement; $f_{a}$ is dynamic output force for piezo actuators;

#### Equivalent modeling of Stewart dynamics and kinematic systems

**Fig. 6:**
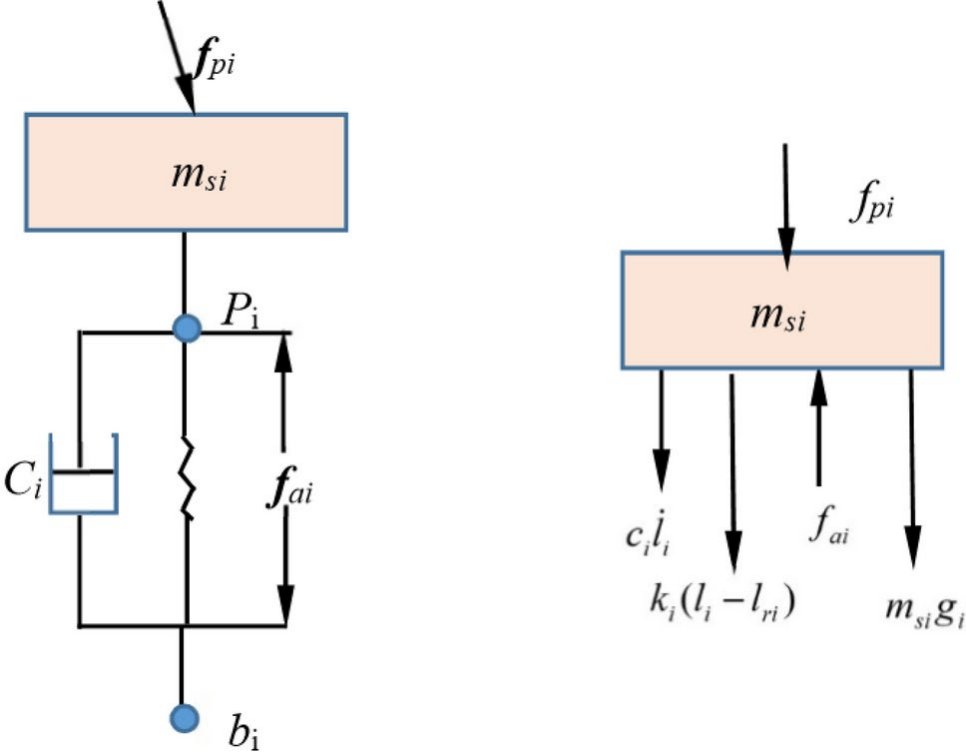
Outrigger mechanics model and force analysis diagram.

The outrigger is modeled according to the spring mass system, and for the $ith$ leg, $m_{i}$ is the mass, $k_{i}$ is the spring stiffness coefficient, $c_{i}$ is the damping coefficient, and $f_{ai}$ is the actuator output force. The force of the ith leg is $f_{pi}$. The mechanical model and force analysis of the first leg is shown in the Fig. 6.

The Newtonian-Euler equation for the legs can be expressed as

17 $$f_{p} = f_{a} - M_{s} \ddot{l} - C\dot{l} - K\Delta l - M_{s} \ddot{b}_{u} - M_{s} g_{u} + M_{s} a_{2}$$

**Fig. 7:**
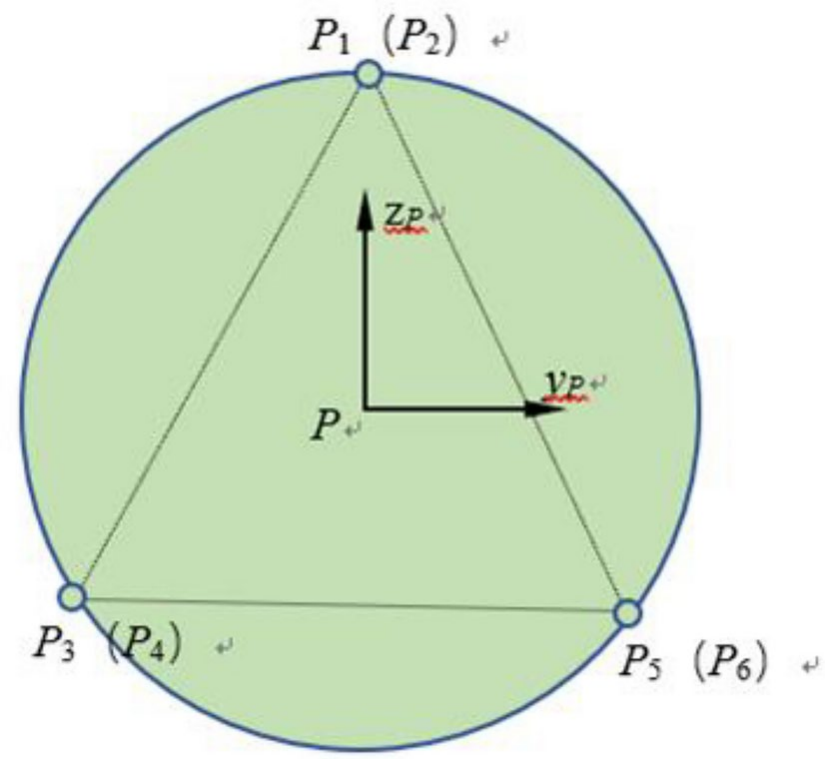
Overhead view of the payload platform.

$g_{u}$ is the component of the acceleration of gravity in the direction of each leg; $a_{2}$ is the relative acceleration; $\ddot{b}_{u}$ Acceleration of the point of articulation of the legs on the star. The action of the 6 outriggers on the load is expressed in terms of principal vector and principal moment. Using the Newtonian-Euler method, the kinetic equations of the load are established as shown in the Fig. 7.

18 $$\left\{ \begin{gathered} m_{p} a_{p} = P_{dp} + P_{lp} + P_{ep} \hfill \\ I_{p} \dot{\omega }_{p} + \tilde{\omega }_{p} I_{p} \omega_{p} = T_{dp} + T_{lp} + T_{ep} \hfill \\ \end{gathered} \right.$$

$P_{dp}$ and $T_{dp}$ are the interference force and interference moment acting on the load center of mass; $P_{ep}$ and $T_{ep}$ are the control force. The forces and moments of the 6 outriggers for the satellite platform are expressed in $P_{lp}$ and $T_{lp}$, and the dynamic equation of the satellite platform is considered by considering the role of the flexible attachment.

19 $$\left\{ \begin{gathered} m_{b} a_{b} + \sum\limits_{j = 1}^{n} {B_{tranj} \ddot{\eta }_{bj} } = P_{db} + P_{lb} + P_{eb} \hfill \\ I_{b} \dot{\omega }_{b} + \tilde{\omega }_{b} I_{b} \omega_{b} + \sum\limits_{j = 1}^{n} {B_{rotj} \ddot{\eta }_{bj} } = T_{db} + T_{lb} + T_{eb} \hfill \\ \ddot{\eta }_{bj} + 2\xi_{bj} \Omega_{bj} \dot{\eta }_{bj} + \Omega^{2}_{bj} \eta_{bj} + B_{tranj}^{T} a_{b} + B_{rotj}^{T} \dot{\omega }_{b} = 0 \hfill \\ \end{gathered} \right.$$

$m_{b}$ and $I_{b}$ are the mass and inertia of the astral platform; $a_{b}$ is the platform centroid acceleration; $P_{db}$ and $T_{db}$ are the interference force and interference torque acting on the center of mass of the platform; $P_{eb}$ and $T_{eb}$ are the control force provided for the piezo actuator is equivalent to the main arrow and main moment behind the platform constitution center. $\ddot{\eta }_{bj}$ is the modal coordinate of the jth flexible attachment on the star; N is the order of modal truncation; $B_{tranj}$ and $B_{rotj}$ are the matrix of translational and rotational coupling coefficients of the jth flexible attachment.

Synthesizing the above system of equations and the relationship between dynamics, ignoring the influence of outrigger mass, the dynamic equation of the load can be expressed as when the motion of the star platform is known

20 $$M_{p} J_{M}^{T} \ddot{l} + J^{T} C\dot{l} + J^{T} f_{a} K\Delta l = J^{T} f_{a} + f_{e} - M_{p} J_{a}^{T} \ddot{\chi }_{a}$$

Matrix $J$ represents the conversion relationship between coordinate systems; $\ddot{\chi }_{a}$ indicates the acceleration caused by the motion of the astral platform; $f_{e}$ is the directional control force.

**Fig. 8:**
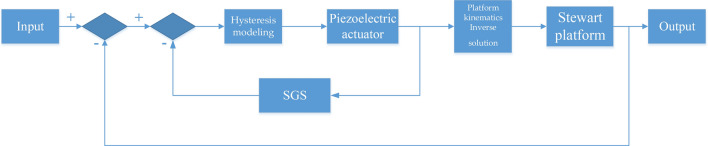
Stewart system control block diagram.

According to the^12^, the sky-hook damper technique can be described as (21), and Stewart system control block diagram is shown in the Fig. 8.

21 $$f_{a} = - gsx$$

## Experiment results and analysis

This experiment includes the following four aspects:

1. Piezoelectric actuator hysteresis inverse compensation
2. Sinusoidal and randomly controlled experiments without cables
3. Sinusoidal control experiment in the failed state of the leg
4. Sinusoidal control experiment in the state of cable
5. 4.1. Hysteresis inverse compensation

With the sky-hook controller, the expression of the active force can be expressed as

22 $$f_{a} = - gsx_{2}$$

The transfer function can be expressed as

23 $$\frac{{x_{2} \left( s \right)}}{{x_{0} (s)}} = \frac{{(c_{1} s + k_{1} )(c_{2} s + k_{2} )}}{{(m_{1} s^{2} + c_{1} s + k_{1} )\left( {m_{2} s^{2} + gs + c_{2} s + k_{2} } \right) + m_{2} s^{2} \left( {c_{2} s + k_{2} } \right)}}$$

**Fig. 9:**
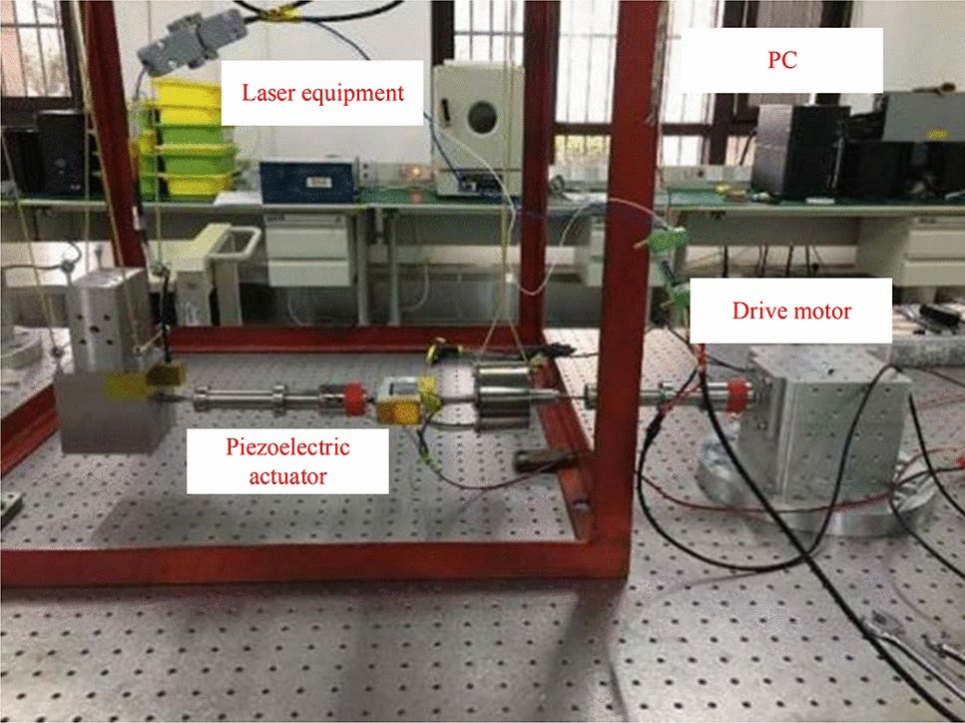
Single rod test diagram.

For parameter identification in the model, the improved MPSO method described above is employed. The identified parameter values are presented in Table 1. To utilize acceleration sensing in the single-leg test diagram, the damper component in the single-leg test setup uses only the spring plate. The control algorithm is implemented using Simulink and Dispace. The piezoelectric actuator used is the PI-330. Upon receiving the acceleration signal, it undergoes bandpass filtering followed by high-pass filtering after integration, as illustrated in Fig. 9.

**Table 1:** Parameter identification in the model.

Parameter	Value
k	5.012 × 10^–6^
α	0.41
A	− 3.571 × 10^–6^
β	0.22
λ	− 0.016
m_1_	0.25 kg
m_2_	60 kg
k_1_	10,000
k_2_	4 × 10^8^
c_1_	70
c_2_	0

**Fig. 10:**
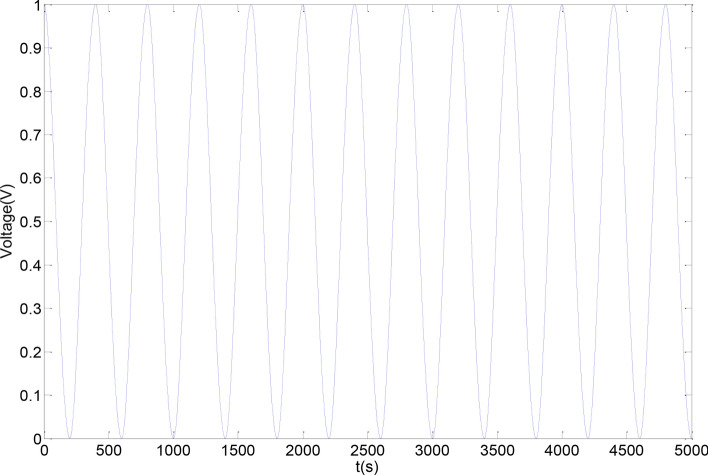
Piezoelectric actuator input signal.

**Fig. 11:**
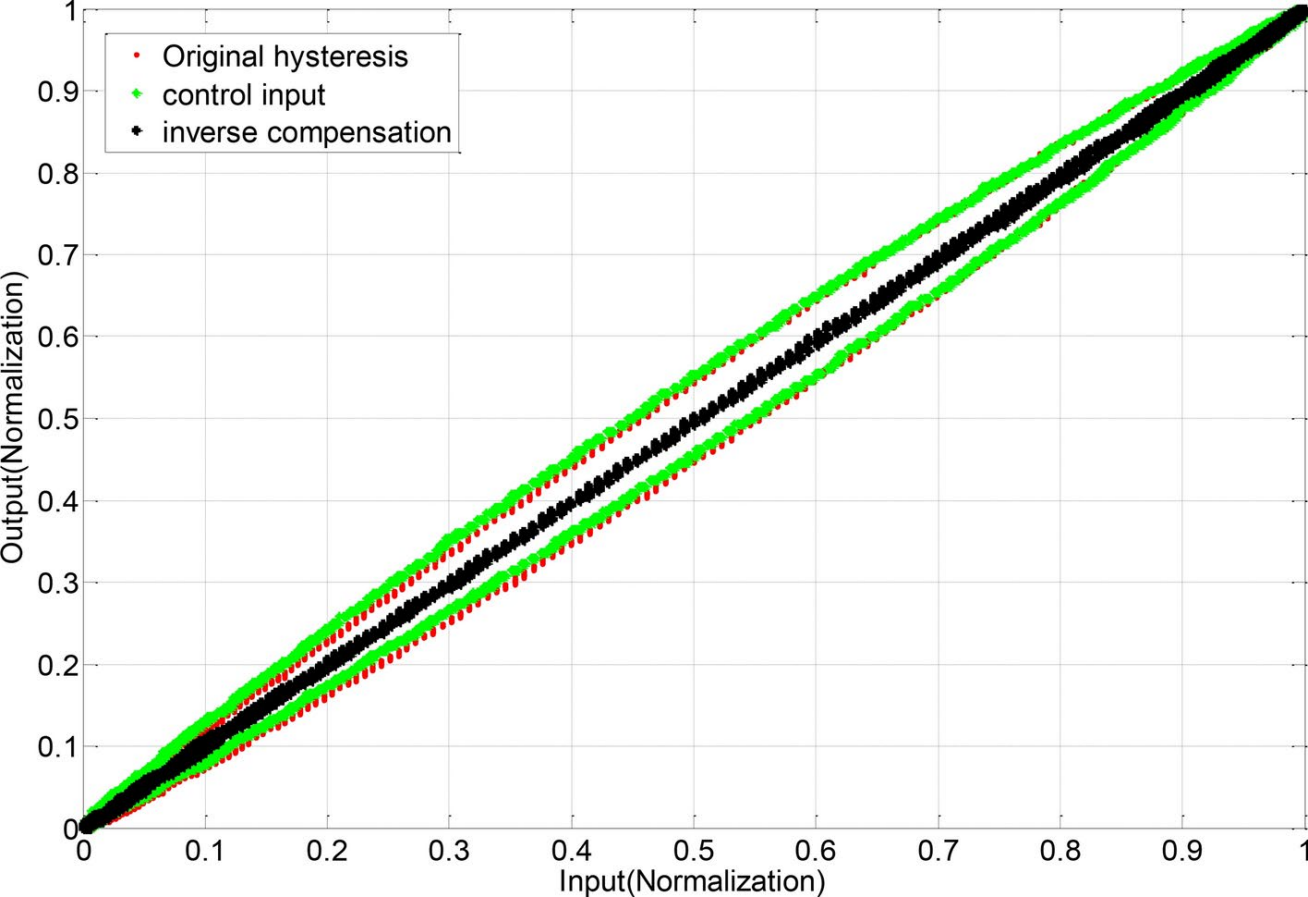
Comparison curve of piezoelectric actuator before and after compensation.

Before applying feed-forward linearization compensation, the average linearity is 8.9% and the hysteresis error is less than 7.79%, indicating a pronounced hysteresis effect. After implementing feed-forward linearization compensation, the average linearity improves to 1.8%, and the hysteresis error is reduced to less than 0.6%. This demonstrates a significant improvement in both linearity and reduction of the hysteresis effect as shown in the Figs. 10 and 11.

### Sinusoidal and randomly controlled experiments without cables

**Fig. 12:**
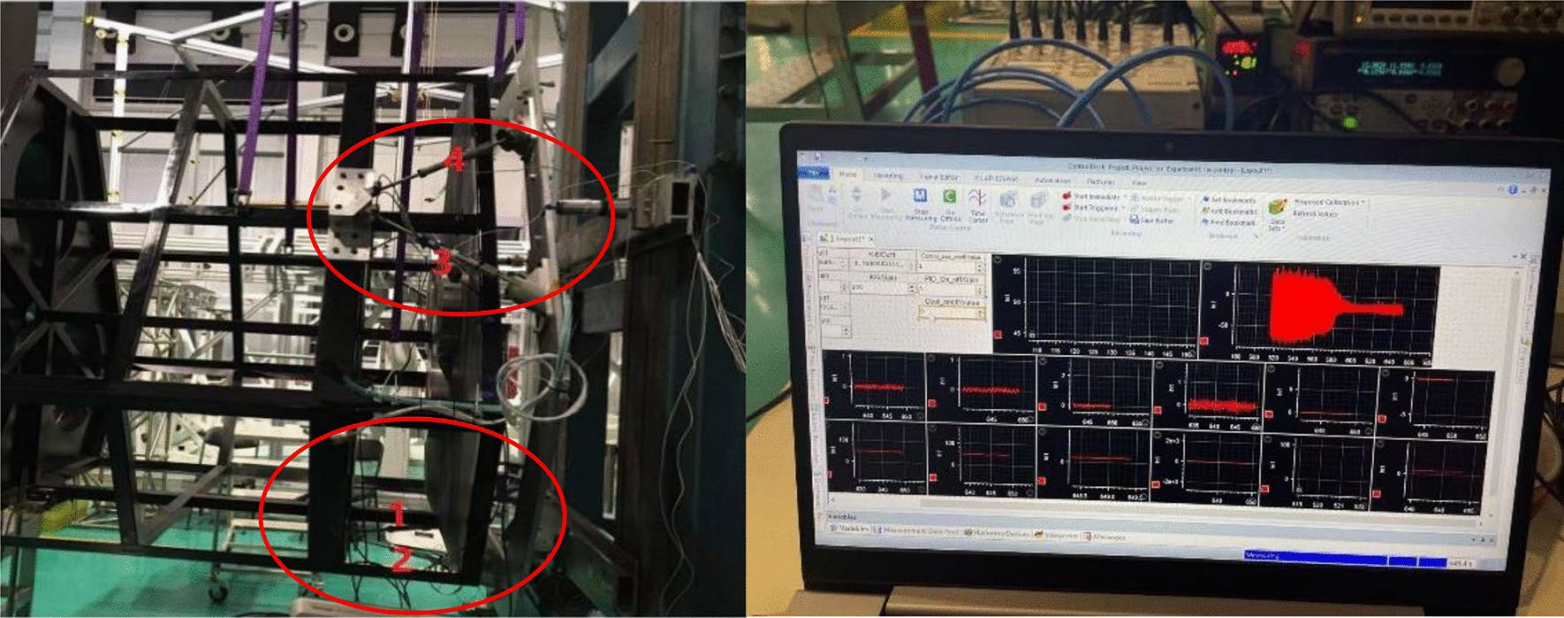
Inverse compensation.

**Fig. 13:**
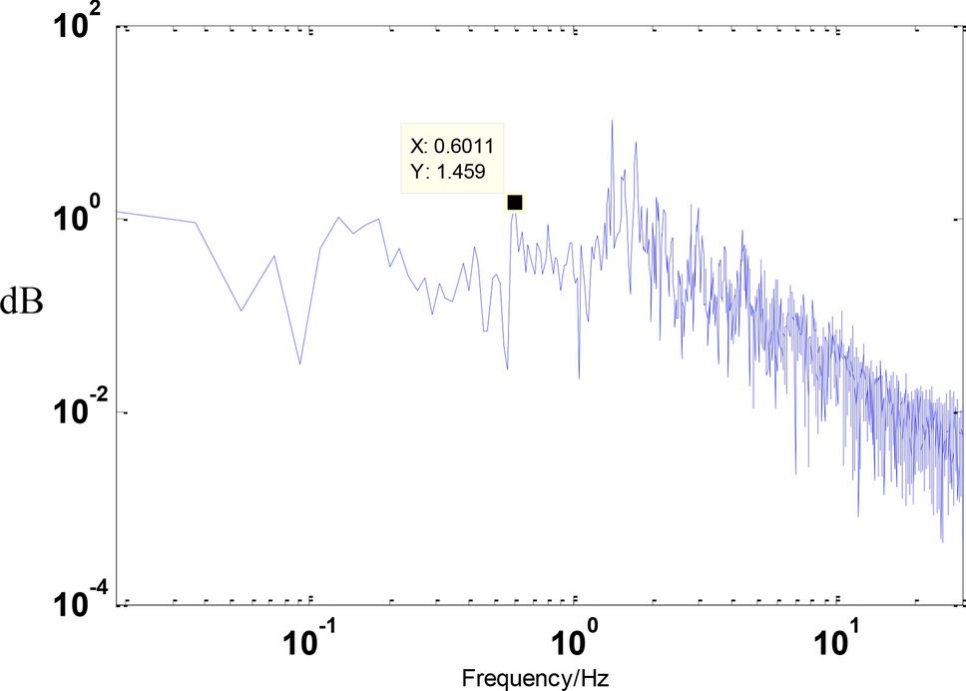
Weep the frequency curve.

Before the experiment, stochastic excitation analysis of the displacement response spectrum was conducted to identify the natural frequency points of the entire vibration isolation system. The results are shown in the figure below. It can be observed from the figure that the first four dominant frequency points are approximately 0.6011 Hz, 1.403 Hz, 1.567 Hz, and 1.73 Hz as shown in the Figs. 12 and 13.

**Fig. 14:**
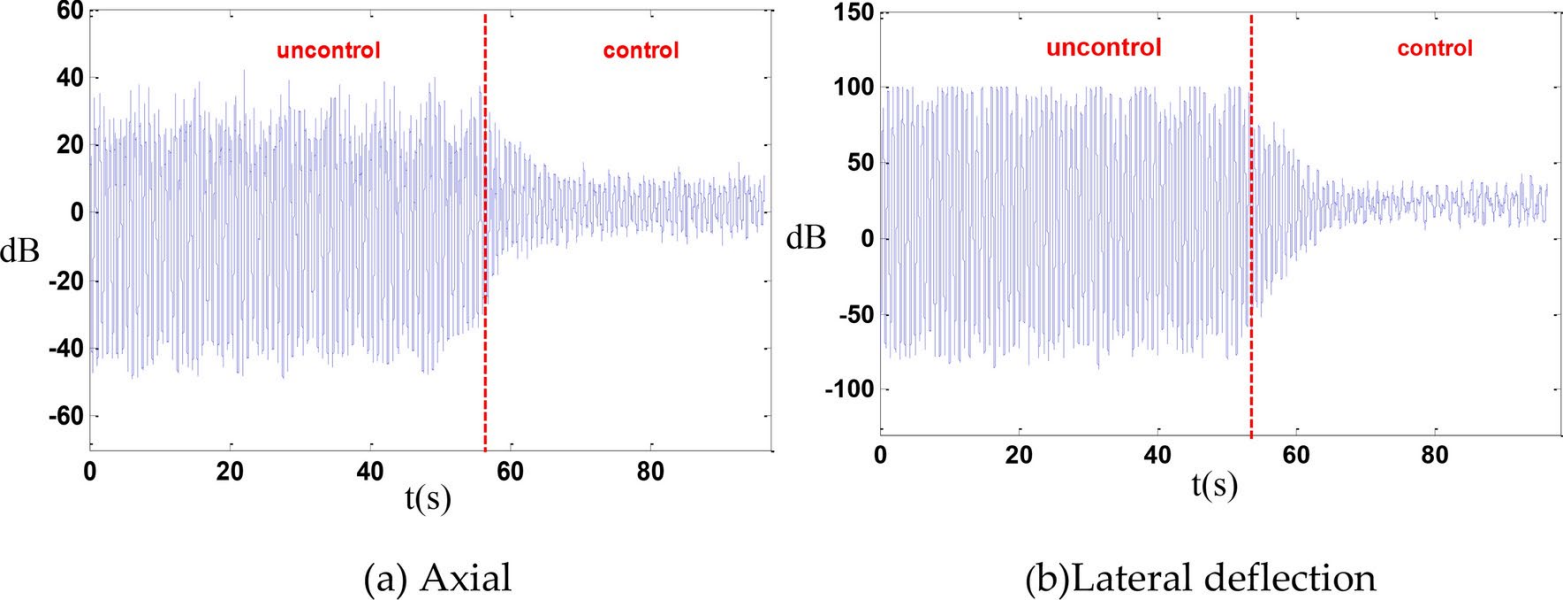
whole machine vibration isolation control.

An excitation frequency of 1.403 Hz was selected for the vibration isolation control of the entire system. After implementing the control, the vibration was reduced by 14 dB in the axial direction and by 15.3 dB in the lateral deflection direction as shown in the Fig. 14.

**Fig. 15:**
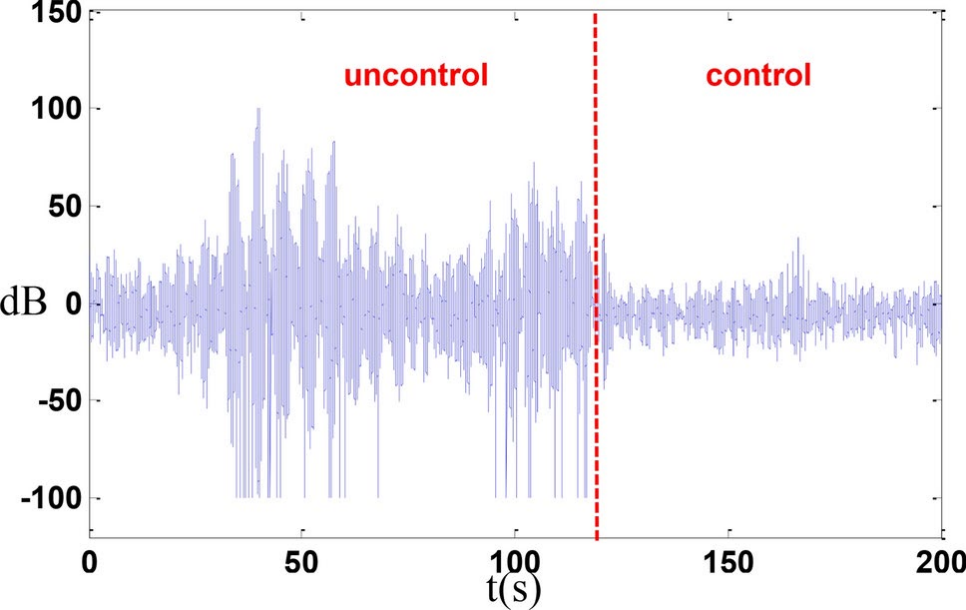
Random control.

In the absence of cables, an axial random control experiment was conducted, resulting in a reduction of axial vibration by 10 dB as shown in the Fig. 15.

### Sinusoidal control experiment in the failed state of the leg

- One leg fails

**Fig. 16:**
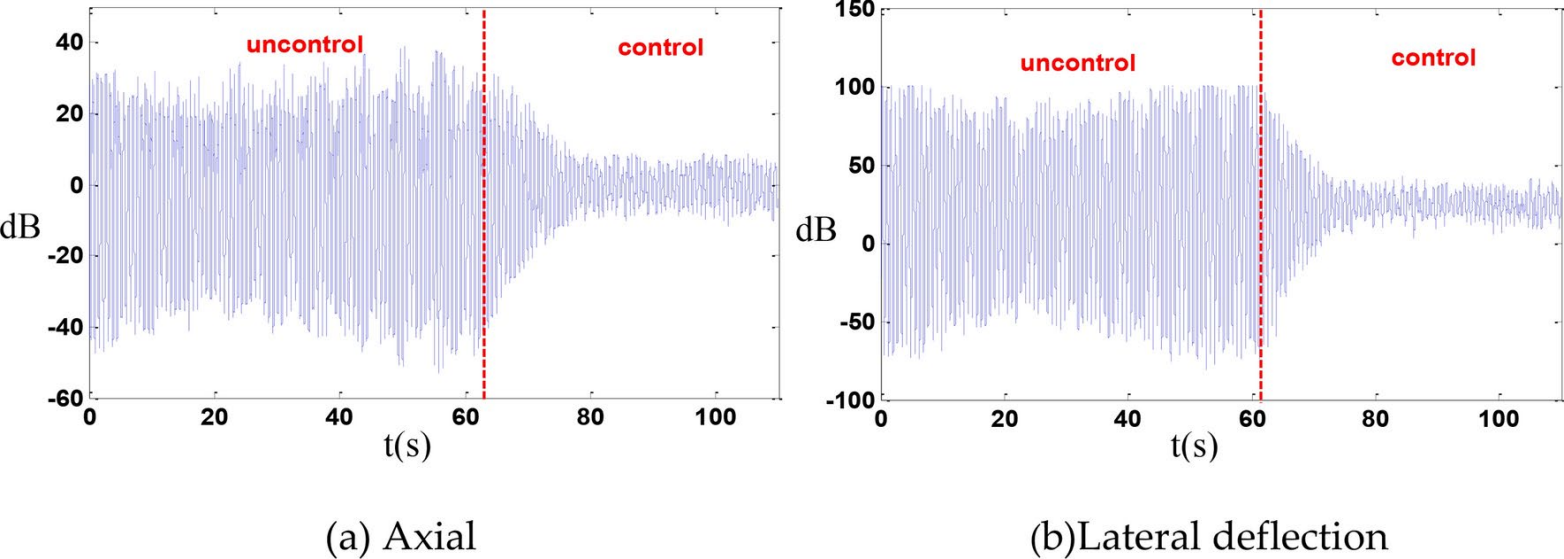
Failure of a single leg.

In this experiment, it was assumed that leg No. 1 fails, and the remaining 5 legs are used for control. Under these conditions, the axial displacement was reduced by 13 dB, and the lateral deflection displacement was reduced by 15.6 dB as shown in the Fig. 16.

b. Both legs fail

**Fig. 17:**
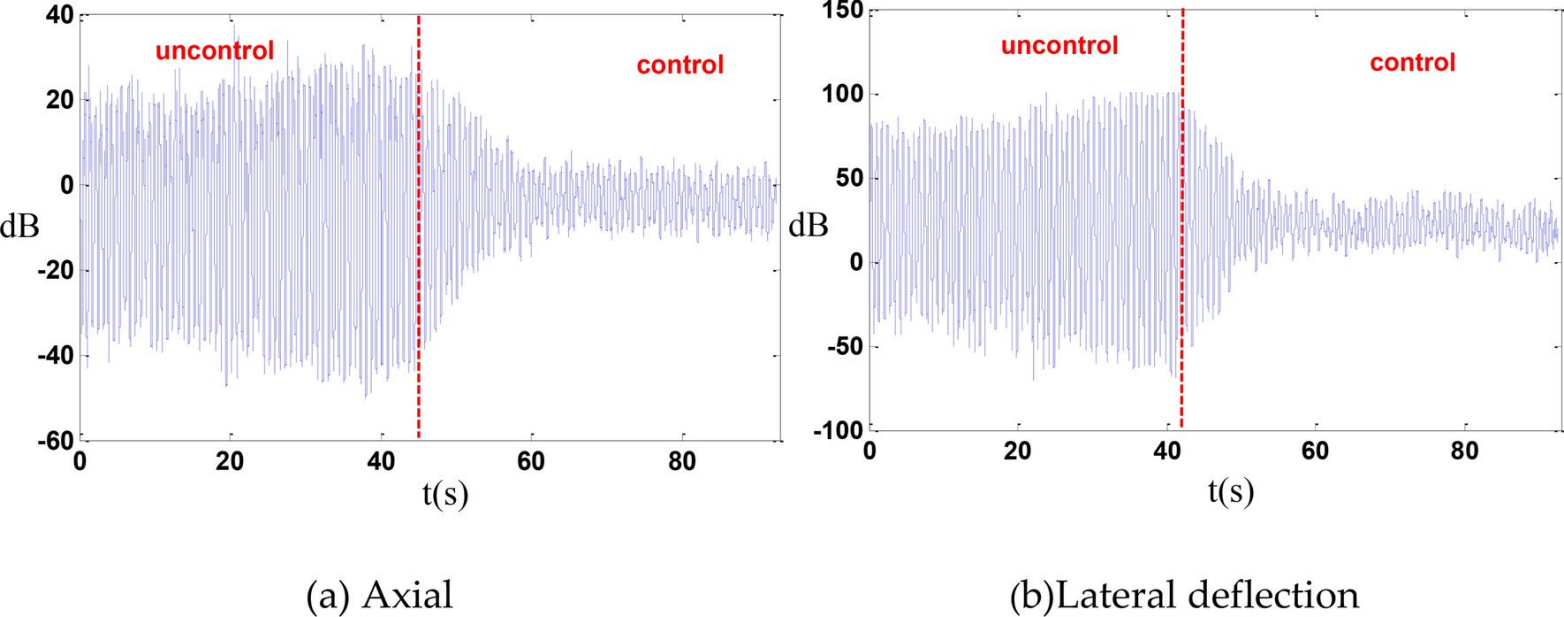
Both legs fail.

Outriggers 1 and 2 can reduce axial displacement by 13.3 dB and lateral deflection displacement by 13.8 dB as shown in the Fig. 17.

c. Three legs fail

**Fig. 18:**
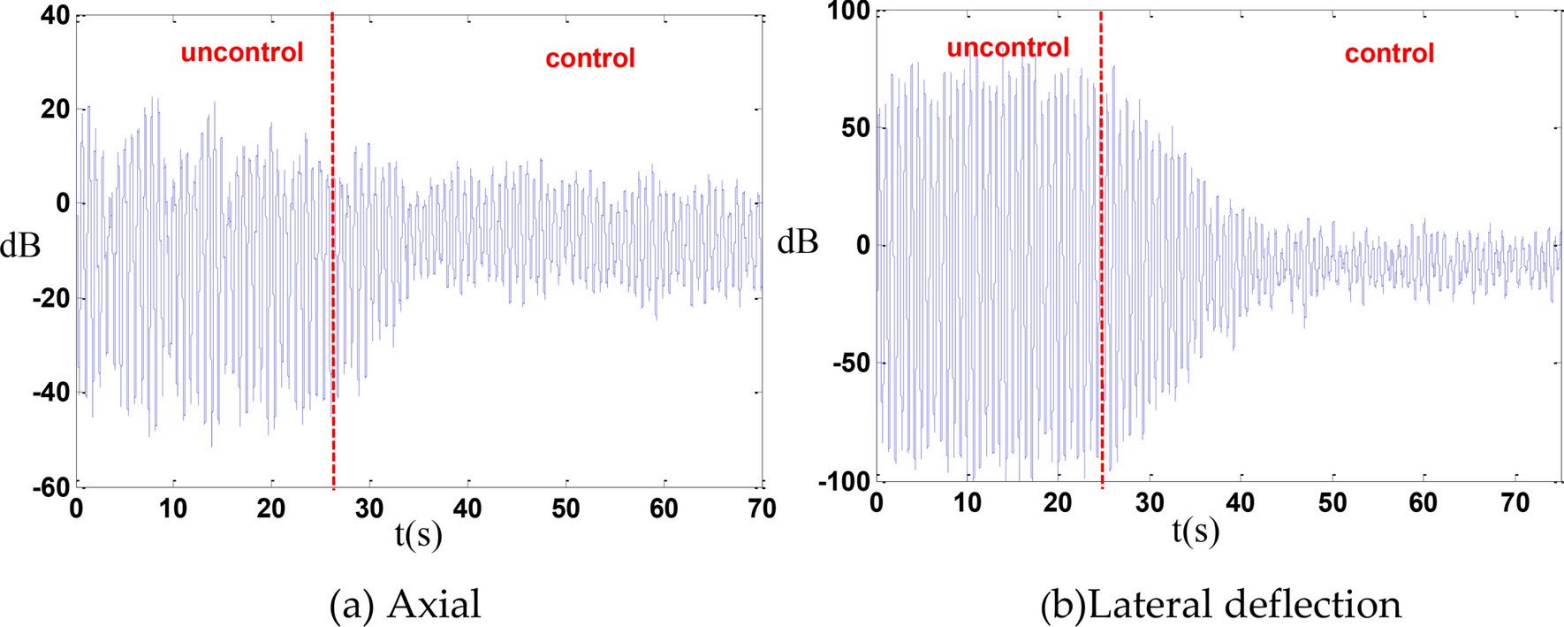
Three legs fail.

When legs 1, 2, and 3 fail, the axial displacement can be reduced by 8.2 dB, while the transverse deflection displacement can be reduced by 14.2 dB as shown in the Fig. 18.

### Sinusoidal control experiment in the state of cable

A. The cable is slightly loose

**Fig. 19:**
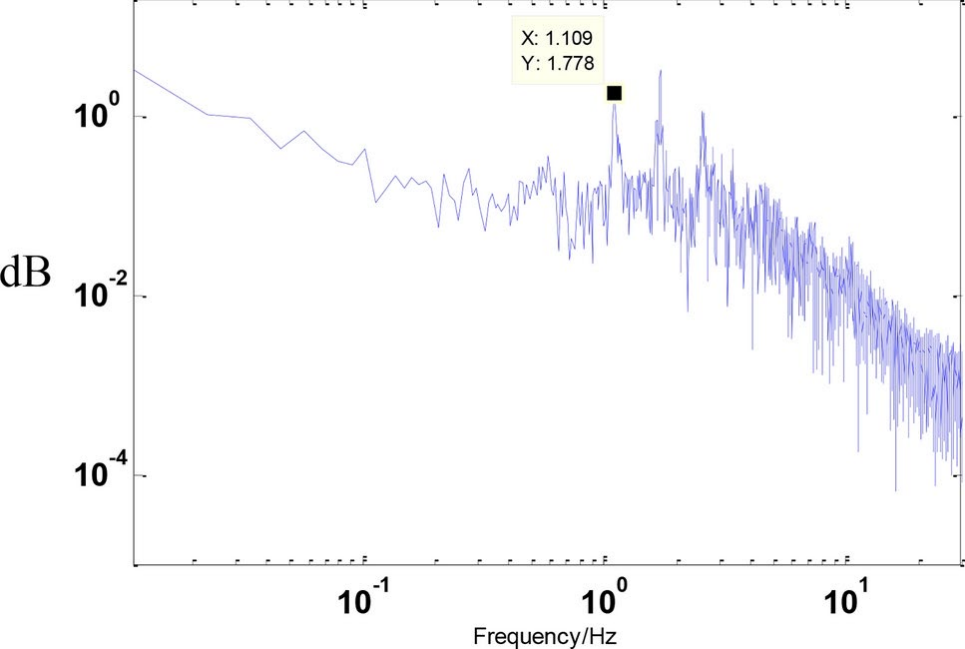
Displacement response spectrum.

Firstly, the natural frequency characteristics of the vibration isolation system were obtained through random excitation analysis of the displacement response spectrum. The primary frequencies for the first three modes were found to be 1.1 Hz, 1.7 Hz, and 2.56 Hz. Compared to the cable-free state, these frequencies are higher, indicating that the addition of cables increases the connection stiffness of the vibration isolation system as shown in the Fig. 19.

**Fig. 20:**
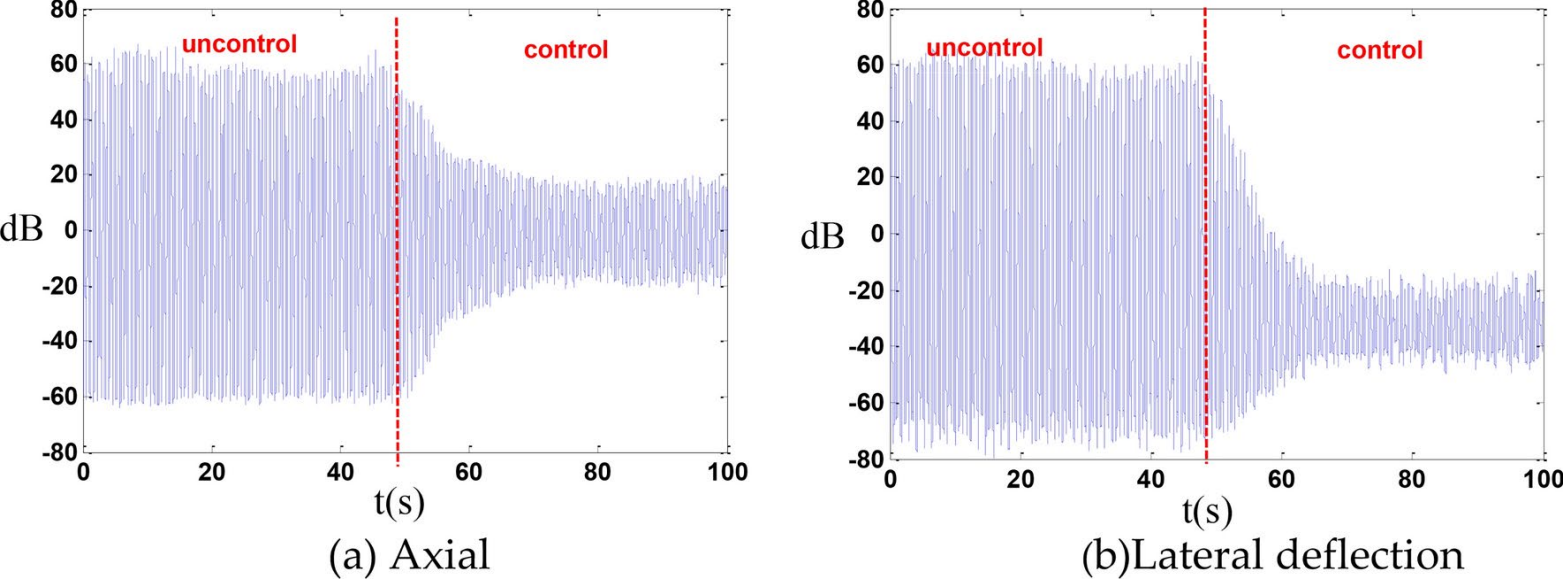
The cable is slightly loose.

In the vibration control experiment, with a main frequency of 1.7 Hz as the excitation frequency, the axial displacement was reduced by 10.7 dB, and the transverse deflection displacement was reduced by 12.6 dB as shown in the Fig. 20.

B. The cable is slightly tight

**Fig. 21:**
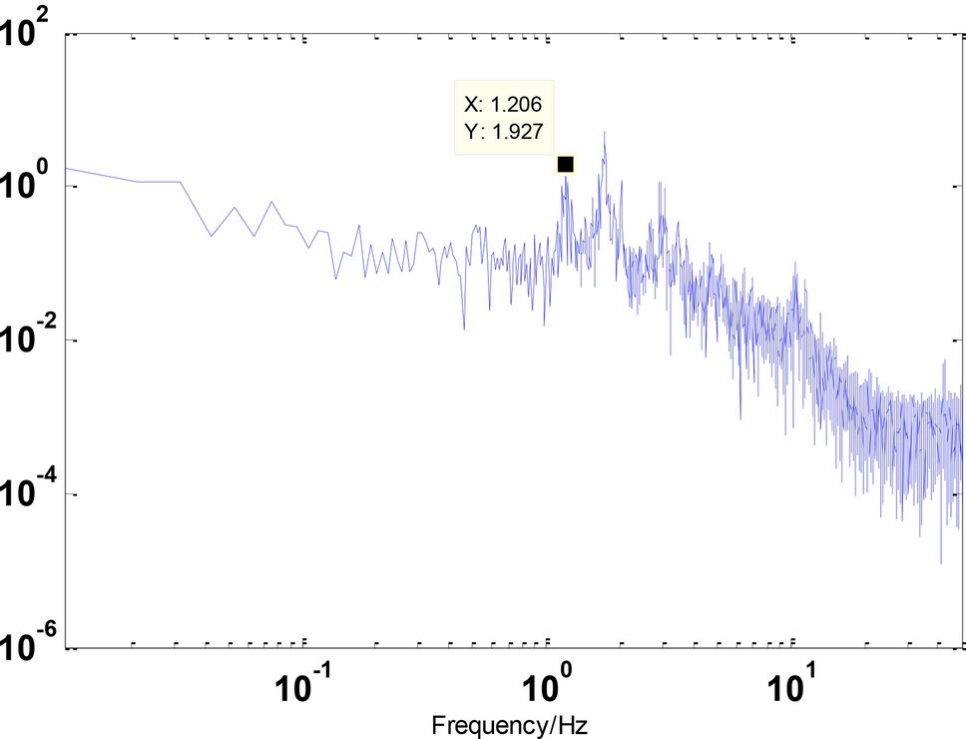
Displacement response spectrum.

First, the natural frequency characteristics of the vibration isolation system were determined using random excitation analysis of the displacement response spectrum. The primary frequencies for the first three modes were 1.2 Hz, 1.73 Hz, and 2.9 Hz. Compared to the initial cable state, these frequencies are higher, indicating that the cables have become tighter and stiffer as shown in the Fig. 21.

**Fig. 22:**
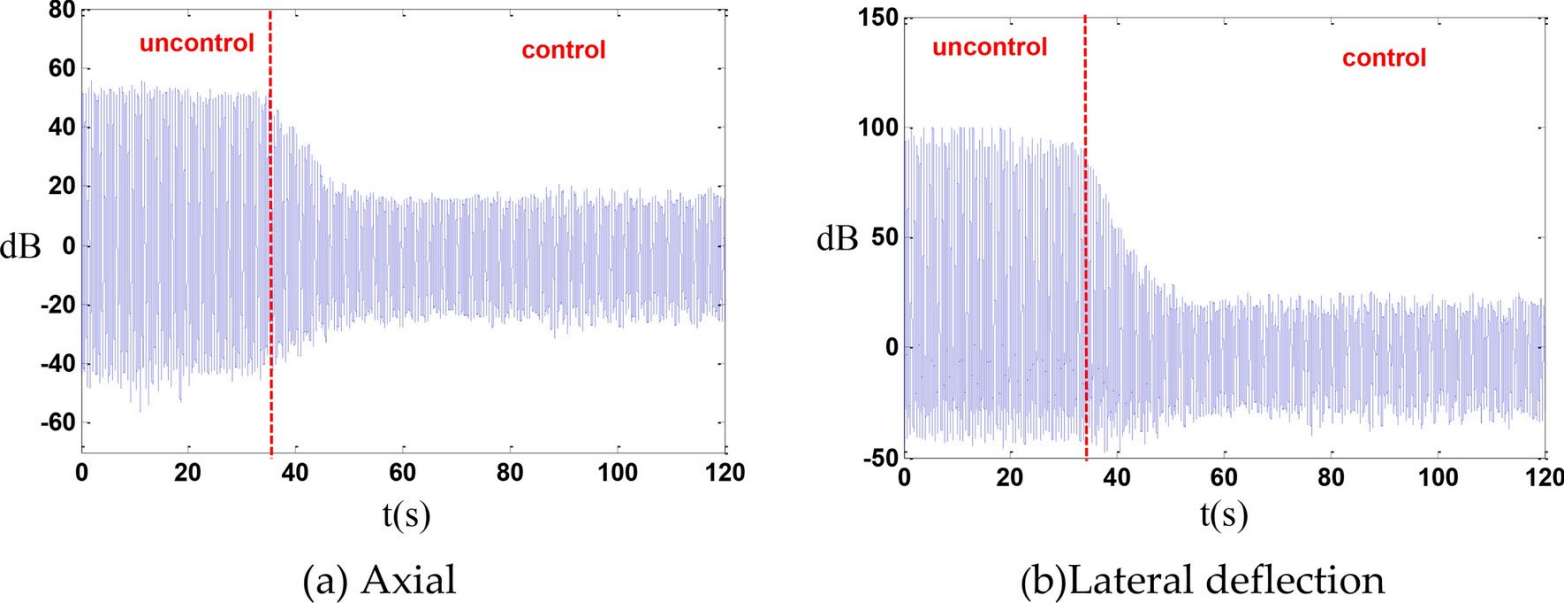
The cable is slightly tight.

In the vibration control experiment, with a main frequency of 1.73 Hz as the excitation frequency, the axial displacement was reduced by 7.0 dB, and the transverse deflection displacement was reduced by 9.0 dB as shown in the Fig. 22.

## Conclusions

In this paper, a vibration isolation control system based on a Stewart platform with six degrees of freedom and hysteresis inverse compensation for piezoelectric actuators is designed, achieving a linearity improvement of over 90%. The system modeling and control method design for the Stewart platform were conducted, demonstrating that the six-degree-of-freedom vibration isolation system remains effective even in the case of outrigger failure. Furthermore, since the degree of cable tension affects the inherent characteristics of the vibration isolation system, it is confirmed that active vibration isolation remains effective by testing cables with varying levels of tension.

## Data Availability

The datasets generated and/or analysed during the current study are not publicly available due [REASON WHY DATA ARE NOT PUBLIC] but are available from the corresponding author on reasonable request.

## References

[1] Yang, L. et al. A high-performance, flexible, and dual-modal humidity-piezoelectric sensor without mutual interference. *Sens. Actuators B Chem.* **423**, 136778 (2024).

[2] Wu, Z. et al. Inversion-based fuzzy adaptive control with prespecifiable tracking accuracy for uncertain hysteretic systems. *Fuzzy Sets Syst.* **498**, 109141 (2025).

[3] Al-Rawashdeh, Y. M. et al. Model-free control for an industrial long-stroke motion system with a nonlinear micropositioning actuator. *Mechatronics* **104**, 103257 (2024).

[4] Stirbu, R. S. & Mitoseriu, L. Modeling of hysteretic response of porous piezo/ferroelectric ceramics. *Comput. Mater. Sci.* **232**, 112633 (2024).

[5] Yuan, Z. et al. Piezo-actuated smart mechatronic systems: Nonlinear modeling, identification, and control. *Mech. Syst. Signal Process.* **221**, 111715 (2024).

[6] Sima, J. et al. Coupled hysteresis and resonance control of three-degree-of-freedom tip-tilt-piston piezoelectric stage. *Precis. Eng.* **89**, 393–407 (2023).

[7] Yin, R. et al. Characterizing the electric field-and rate-dependent hysteresis of piezoelectric ceramics shear motion with the Bouc–Wen model. *Sensors Actuators A Phys.* **367**, 115044 (2024).

[8] Lu, M. et al. Compensation control strategy of hybrid driven three-dimensional elliptical vibration assisted cutting system based on piezoelectric hysteresis model. *Meas. Control* **57**(3), 319–329 (2024).

[9] Li, X. et al. Data-driven koopman learning and prediction of piezoelectric tube scanner hysteresis. *IEEE Trans. Syst. Man Cybern. Syst.* **54**, 3631–3641 (2023).

[10] He, M. et al. Non-singular fast terminal sliding mode trajectory tracking control of cantilever piezo-electric stack actuator based on asymmetric hysteresis compensation. *Smart Mater. Struct.* **34**, 015013 (2023).

[11] Zhou, M. et al. Modeling, identification, and high-speed compensation study of dynamic hysteresis nonlinearity for piezoelectric actuator. *J. Intell. Mater. Syst. Struct.* **35**(9), 822–844 (2024).

[12] Shvetsov, I. A. et al. Piezoelectric hysteresis and relaxation processes in ferroelectric ceramics in weak electric fields. *Bull. Rus. Acad. Sci. Phys.* **88**(5), 709–714 (2024).

[13] Hao, G. et al. Rate-dependent hysteresis modeling and compensation for fast steering mirrors. *Sensors Actuators A Phys.* **376**, 115568 (2024).

[14] Nguyen, T. T. et al. Nonlinear modeling of a piezoelectric actuator-driven high-speed atomic force microscope scanner using a variant densenet-type neural network actuators. *MDPI* **13**(10), 391 (2024).

[15] Son, N. N., Van Kien, C. & Anh, H. P. H. Parameters identification of Bouc–Wen hysteresis model for piezoelectric actuators using hybrid adaptive differential evolution and Jaya algorithm. *Eng. Appl. Artif. Intell.* **87**, 103317 (2020).

[16] Chaudhary, S., Sharma, A. & Singh, V. Optimization of high speed and long-haul inter-satellite communication link by incorporating differential phase shift key and orthogonal frequency division multiplexing scheme. *Optics* **176**, 185–190 (2019).

[17] Whba, R. et al. Intrinsic challenges and strategic approaches for enhancing the potential of natural rubber and its derivatives: A review. *Int. J. Biol. Macromol.* **276**, 133796 (2024). 39004255 10.1016/j.ijbiomac.2024.133796

[18] Li, R., Cao, K. & Li, Z. Modelling and compensation of rate-dependent hysteresis in piezoelectric actuators based on a modified Madelung model. *Electron. Lett.* **60**(18), e70027 (2024).

[19] Shao, S. B. et al. Structure and controller design of a piezo-driven orientation stage for space antenna pointing. *Mech. Syst. Signal Process.* **138**, 106525 (2020).

[20] Lu, K. et al. Finite-time prescribed performance tracking control for nonlinear time-delay systems with state constraints and actuator hysteresis. *ISA Trans.* **153**, 295–305 (2024). 39117473 10.1016/j.isatra.2024.07.027

[21] Hu, W. et al. Effects of pedicle subtraction osteotomy on aortic morphology and hemodynamics in ankylosing spondylitis with kyphosis: A finite element analysis study. *Sci. Rep.* **14**(1), 25456 (2024). 39462112 10.1038/s41598-024-77417-3 PMC11512994

[22] Ajin, R. S., Segoni, S. & Fanti, R. Optimization of SVR and CatBoost models using metaheuristic algorithms to assess landslide susceptibility. *Sci. Rep.* **14**(1), 24851 (2024). 39438526 10.1038/s41598-024-72663-x PMC11496660

[23] Wang, X. et al. An enhanced micro-PSO method to deal with asymmetric electricity markets competition within hydropower cascade. *Appl. Energy* **377**, 124235 (2025).

[24] Öztürk, E., Çavdar, A. D. & Çavdar, T. Optimization of mixture ratios of raw materials in thermoplastic hybrid composites based on particle swarm optimization algorithm. *J. Supercomput.* **81**(1), 13 (2025).

[25] Yang, Y. et al. Optimization method of vibration isolation performance of the 6DOF vibration isolation platform based on different configurations. *Int. J. Non-Linear Mechanics* **167**, 104924 (2024).

[26] Jiang, M. et al. Control and experimental study of 6-DOF vibration isolation platform with magnetorheological damper. *Mechatronics* **81**, 102706 (2022).

[27] Liu, Y. et al. Force transmissibility of a 6-DOF passive quasi-zero stiffness vibration isolation platform. *J. Mech. Sci. Technol.* **35**(6), 2313–2324 (2021).

[28] Nair, V. G., Hegde, N. T. & Dileep, M. V. Active vibration isolation using tilt horizontal coupling immune inertial double link sensor for low frequency applications. *J. Robot. Control* **5**(6), 1673–1689 (2024).

[29] Li, J. et al. Modeling of a six-degree-of-freedom magnetic levitation platform actuated by noncontact Lorentz forces. *J. Vib. Control*10.1177/10775463241273035 (2024).

[30] Nguyen, T. N. et al. A high-precision active vibration isolation control system: Experimental study. *Appl. Sci.* **14**(17), 7966 (2024).

